# Rotator Cuff Repair by All-Arthroscopic Versus Mini-Open Technique: A Comparison of Clinical and Functional Outcome

**DOI:** 10.7759/cureus.71546

**Published:** 2024-10-15

**Authors:** Saurabh Daga, Mahak Baid, Pushpal Sarkar, Ayon Das, Rahul Hemant Shah, Karthikeyan Dhandapani

**Affiliations:** 1 Orthopaedics, University Hospitals Birmingham NHS Foundation Trust, Birmingham, GBR; 2 Orthopaedics and Traumatology, Aneurin Bevan University Health Board, Newport, GBR; 3 Orthopaedics, Swansea Bay University Health Board, Swansea, GBR; 4 Orthopaedics, Employees’ State Insurance (ESI) Post Graduate Institutes for Medical Sciences and Research (PGIMSR) Employees’ State Insurance Corporation (ESIC) Medical College and Hospital, Joka, Kolkata, IND; 5 Orthopaedics, Aneurin Bevan University Health Board, Newport, GBR

**Keywords:** arthroscopic repair, mini open repair, rotator cuff tear, shoulder joint, ucla score

## Abstract

Background: Rotator cuff tears can lead to debilitating shoulder function and impairment. Rotator cuff repair aims to eliminate pain and improve function with increased shoulder strength and range of motion. This study evaluated the differences between all-arthroscopic (AA) and mini-open (MO) repair procedures for rotator cuff tendon tears regarding clinical and functional outcomes.

Methods: This was a prospective study conducted at a tertiary care government Hospital in Kolkata, India, between March 2015 and September 2016 which evaluated 25 patients who had undergone all-arthroscopic surgery and 25 patients who underwent mini-open repair (total of 50 patients) for rotator cuff tear with a minimum one-year follow-up. The UCLA score was used to assess the functional outcome of these patients.

Results: The mean age of all patients included in this study was 45.32 years. 86% of patients were male. The two groups had similar demographic characteristics, pre-operative baseline parameters, and intra-op findings. The average UCLA score showed significant improvement from 13.92 pre-operatively to 29.76 at the final follow-up. The patients in the all-arthroscopic surgery group experienced a notable decrease in pain within the first three months compared to the mini-open group. However, at the time of the final follow-up, no significant difference was noted between both groups when comparing the University of California at Los Angeles (UCLA) score, Visual Analogue Scale (VAS) score for pain, and active or passive glenohumeral motion.

Conclusion: The outcomes of all-arthroscopic and mini-open rotator cuff repair surgery are equivocal in terms of both clinical and functional results, with no significant difference in post-operative pain, shoulder joint strength range of motion, or patient satisfaction over the long term.

## Introduction

Rotator cuff tears are one of the most common causes of morbidity affecting the shoulder joint [1] and can occur due to traumatic injuries, degenerative overuse, or a combination of both [2]. Apart from pain, it can also cause limitations in the range of motion and functional disability, thereby affecting the quality of life [3,4].

Rotator cuff tears are common among the elderly and athletes. Manual labourers in India who frequently lift heavy objects are more susceptible to rotator cuff injuries. A fall, blow to the shoulder, or other traumatic injury can result in a full or partial-thickness tear of the rotator cuff. Repetitive overuse can result in a tear and chronic degeneration of these muscles. Heavy overhead lifting also increases the risk of a rotator cuff injury.

Optimal treatment for rotator cuff tears in the elderly remains the subject of some controversy [5]. Some authors suggest conservative treatment with physical therapy and exercise to eliminate pain and restore function for mild to moderate tears [6,7]. However, surgical management and proper rehabilitation are usually required to help regain the shoulder's muscle strength, function, and flexibility and relieve the pain so that the patient can participate in demanding activities [8].

Desirable repair of the rotator cuff includes achievement of high fixation strength, minimal gap formation and maintenance of mechanical stability under cyclic loading, and proper healing of tendon to bone. Surgery is usually indicated for full-thickness rotator cuff tears and has better results than conservative treatment [9-11]. Clinical guidelines recommend using open surgery, mini-open surgery, or arthroscopy for a full-thickness tear accessible to direct repair by suture [12].

All-arthroscopic (AA) and mini-open (MO) repairs are the most effective methods for repairing rotator cuff tears [13,14]. Both techniques have been proven to provide excellent and comparable outcomes. However, the AA procedure is a less invasive technique with good cosmetic results, allows easy access to the glenohumeral joint, is associated with less post-operative pain, shorter hospital stays, fewer wound complications, and provides for faster recovery and rehabilitation [15]. However, it has a longer learning curve, a more complex technique, and a higher cost [16,17]. MO repairs produce adequate results associated with smaller skin incisions, less soft tissue dissection, and a decreased chance of deltoid muscle detachment [18-20]. The choice of surgical technique is based on surgeon preference. There is still no consensus on whether any one of the methods offers a superior outcome [21,22].

The objective of this study was to represent our experience comprising cases of rotator cuff tears that were repaired by AA or MO technique and compare their clinical and functional outcome in an Indian scenario.

## Materials and methods

This study was performed after receiving clearance from the ethical committee of the institute (Approval no. MC/KOL/IEC/NON-SPON/408/11-2014). A total of 50 patients presenting with rotator cuff tears in the Department of Orthopaedics, Medical College, Kolkata, India, from March 2015 to September 2016 and fulfilling the inclusion criteria were included in this study. They were distributed randomly in two groups of 25 patients each. One group was surgically managed by repair via the AA technique, and the other group via the MO technique.

Inclusion criteria

The study included patients aged 20 to 65 with a clinically diagnosed full-thickness rotator cuff tear, confirmed by MRI, and a healthy contralateral shoulder.

Exclusion criteria

Patients with a massive tear (>5 cm), associated shoulder pathology like adhesive capsulitis, inflammatory or degenerative arthritis, previous history of fracture around ipsilateral shoulder or surgery, glenohumeral instability, associated superior labral anterior-posterior (SLAP) lesion, or labral tear were excluded.

Operative procedure

The patients were subjected to a thorough history, clinical examination, and pre-operative routine laboratory investigations, supplemented by radiographs in antero-posterior and scapular Y-view of the shoulder joint, along with an MRI.

All-Arthroscopic Technique

Standard shoulder arthroscopy portals were used for the AA technique of rotator cuff repair. The posterior portal was used for visualization of the rotator cuff, while the lateral portal was used as a working portal for subacromial decompression and cuff repair. An extensive subacromial bursectomy was performed, and tendon edges were debrided.

**Figure 1:**
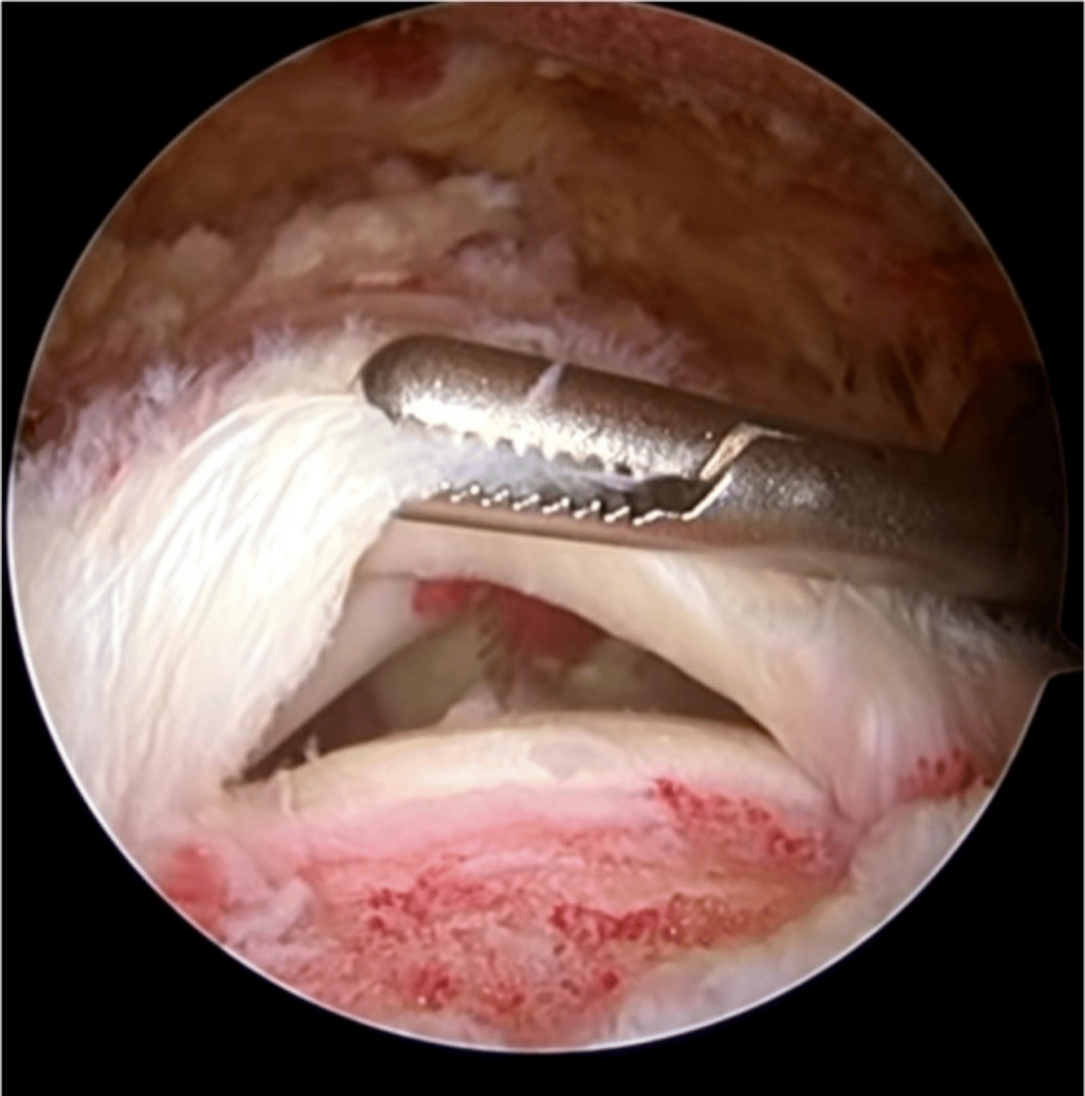
Rotator cuff tear visualized after cuff debridement and mobilization

**Figure 2:**
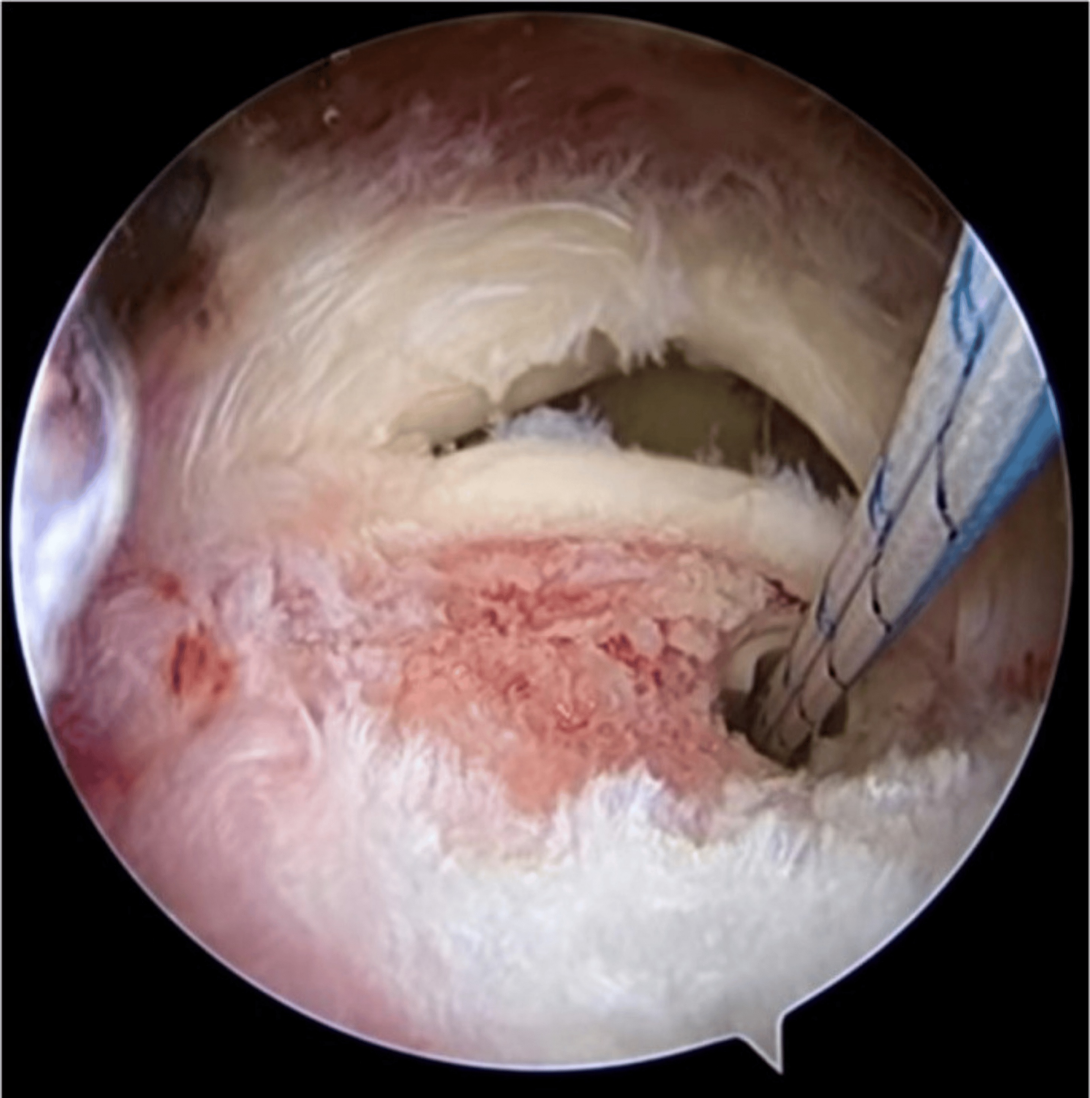
First suture anchor implanted and suture passed through tendon by suture

**Figure 3:**
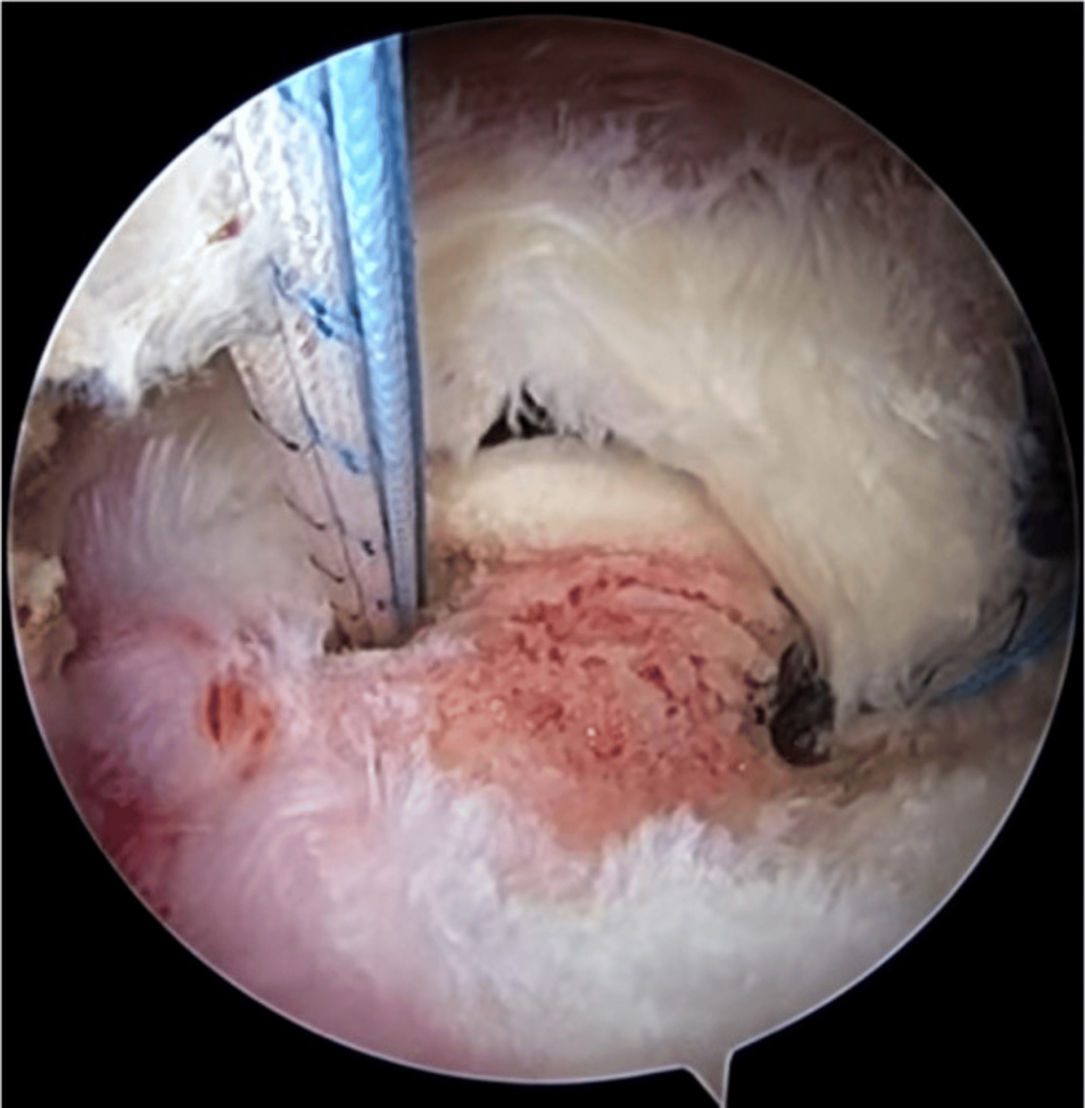
Second suture anchor implanted

**Figure 4:**
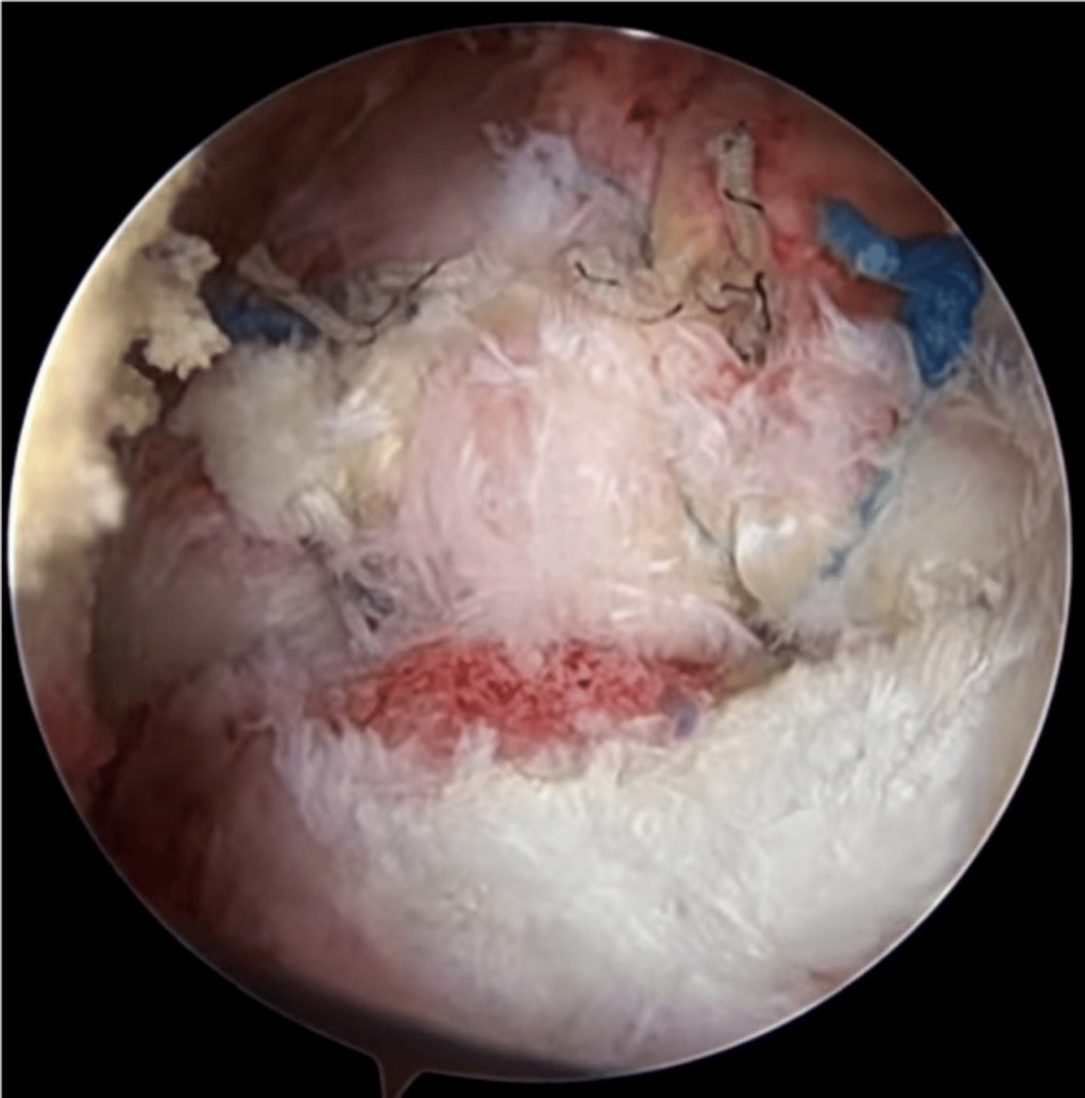
After tying the sutures, tendon is firmly adhered to bone

The size of the rotator cuff tear was assessed using a probe, and the rotator cuff tendon was mobilized so that anatomical reconstruction could take place. Arthroscopic greater tuberosity abrasion was performed using an arthroscopic burr, and one to three suture anchors were placed based on the size of the tear. The repair was undertaken using a single-row technique. An arthroscopic suture passer passed anchor sutures through the tendon in a simple stitch manner. All knots were tied with four alternating half-hitches. The repair was inspected by rotating the arm, and surgical closure of portals was done (Figures 1-4).

Mini-Open Technique

All MO procedures were performed with the patient in the beach-chair position. Using standard shoulder anterolateral portals, arthroscopic subacromial inspection and decompression were performed. The rotator cuff tear location and size were documented in all cases. Rotator cuff tear size was measured by comparing it with the arthroscopic probe of known size at the point of the greatest antero-posterior diameter of the tear.

**Figure 5:**
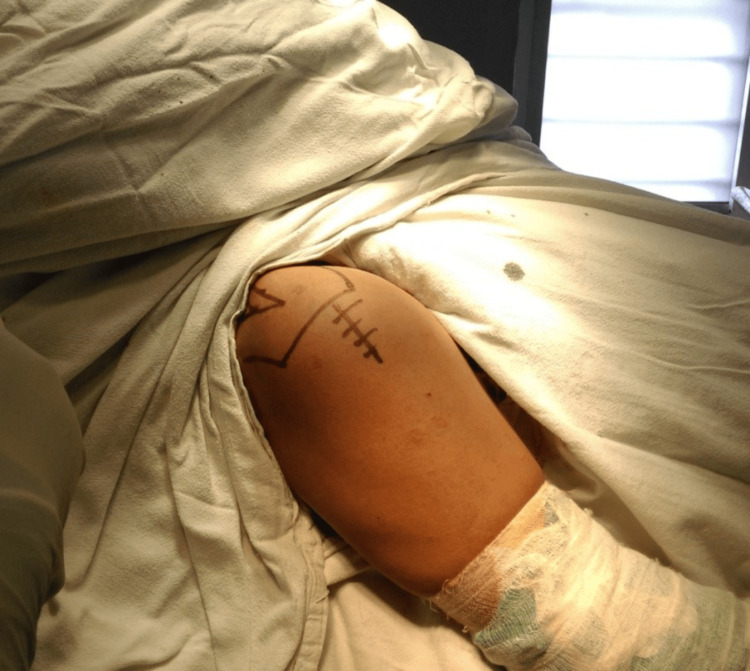
Pre-operative marking over shoulder

**Figure 6:**
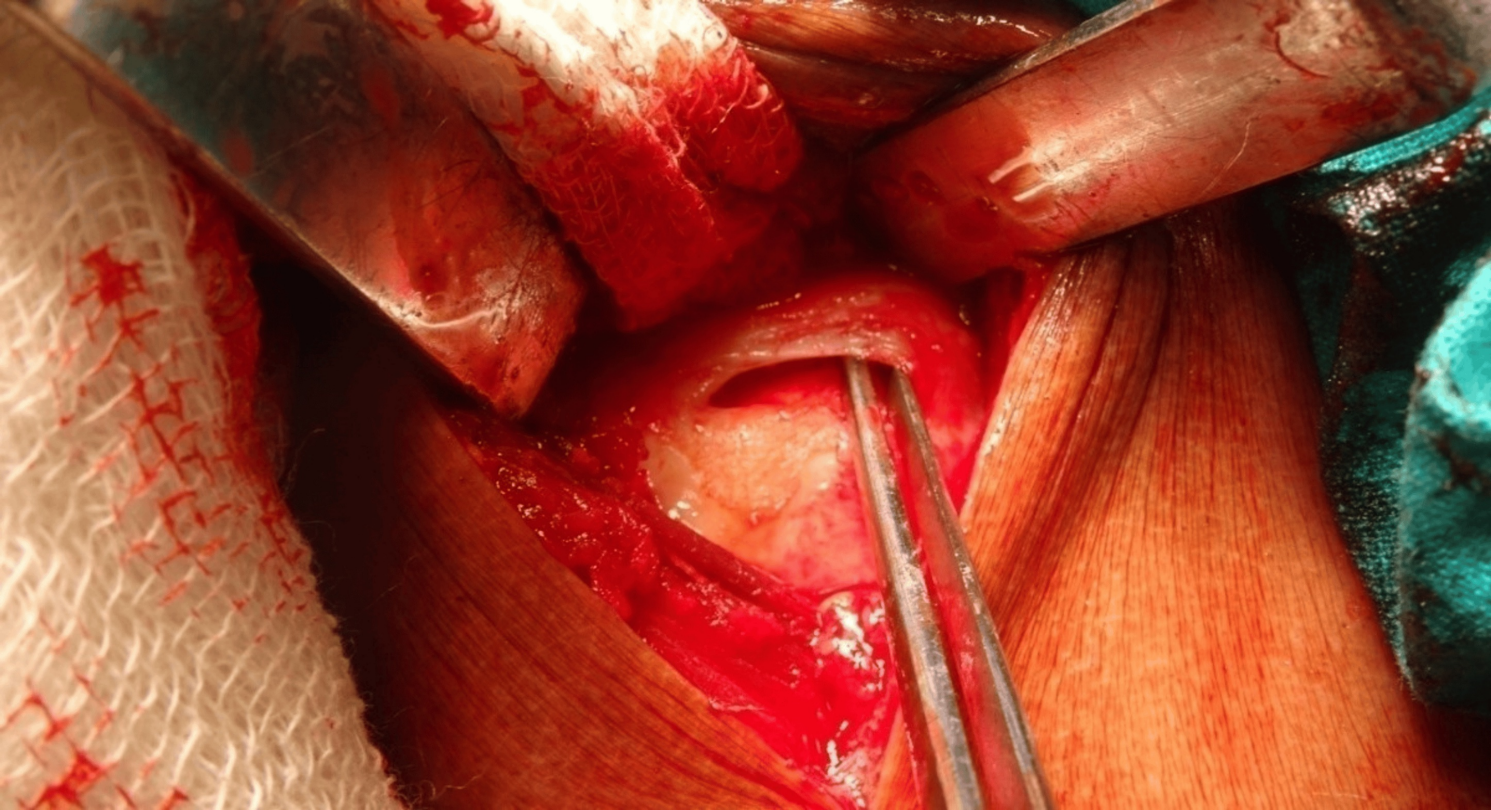
Rotator cuff tear visualized and footprint freshened

**Figure 7:**
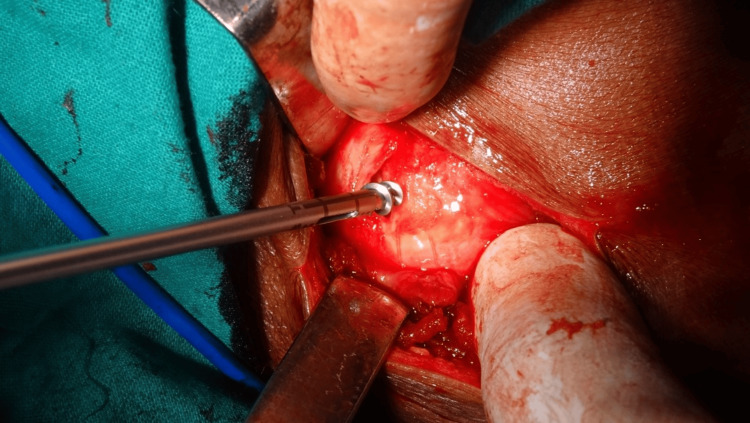
Suture anchor being inserted at the footprint

**Figure 8:**
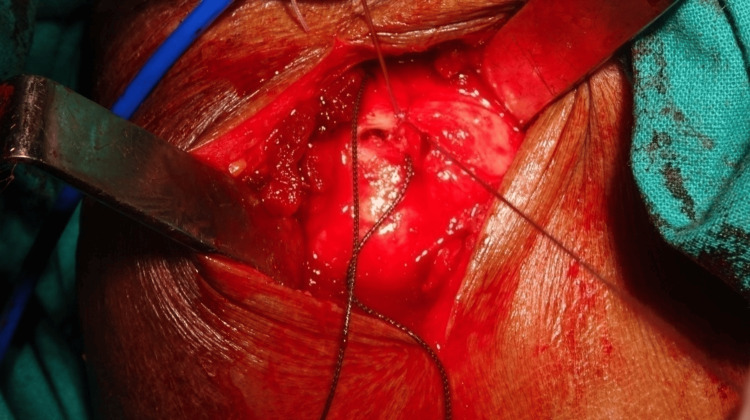
Sutures passed through cuff tissue and margins approximated

The incision for mini-open repair was made by extending the anterolateral portal 3-4 cm downwards. After splitting the deltoid, tears in the cuff were measured again. The greater tuberosity surface was superficially abraded using a burr. The extent of this area abraded was from articular footprint to greater tuberosity. The anterior and posterior dimensions of the abrasion were based on the size of the tear, and one to three suture anchors were placed depending on the size of the tear. In the medial-lateral dimension, the anchors were placed midway between the articular surface and greater tuberosity. A free needle was used to secure the sutures through the tendon with a simple stitch, and all knots were tied with four alternating half-hitches. The arm was internally and externally rotated to inspect the repair, and the deltoid and skin closed in layers (Figures 5-8).

Rehabilitation

Immediately after surgery, the arm was maintained in a sling with 30° abduction in an abduction pillow. Pendulum exercises, scapular mobilization, and passive ROM exercises were initiated during the first week and continued for four weeks. Full shoulder passive ROM in all planes - flexion, abduction, external rotation, and internal rotation - was encouraged, and active assisted ROM exercises were started after four weeks. Sling was discontinued after six weeks. The patients were asked to begin active ROM in forward elevation, external rotation, and abduction by eight weeks. In addition, isometric strengthening exercises were begun at this time. After 12 weeks, the patients underwent resisted exercises using an elastic band or hand weights. Scapular muscle strengthening exercises were also introduced. Gradual return to strenuous work, recreational activities, and sports were allowed after 24 weeks based on the strength and ROM achieved.

The patients were regularly followed up for one year at two weeks, four weeks, three months, six months, and one-year intervals. During the first visit at two weeks, only ROM was addressed, and suture removal was done. Subsequent visits comprised a thorough clinical, radiological, and functional examination. Clinical assessment included pain measurement using the Visual Analogue Scale (VAS) score and estimation of shoulder ROM. Radiological assessment was done by antero-posterior and scapular Y-view radiographs of the shoulder. Functional outcome was appraised using the UCLA (University of California at Los Angeles) score.

Statistical analysis

The statistical software SPSS version 20 was used for analysis (IBM Corp., Armonk, NY). Descriptive analysis was performed for patient demographics and all variables. Pre- and post-outcome scores, range of motion, and pain scale were compared using Students’s paired t-tests, Pearson chi-square test, and Fischer’s exact test to determine whether there were any significant differences between proportions. Significance (p-value) was set at 0.05.

## Results

A total of 50 patients were considered who met the study inclusion criteria. The study population was randomly distributed in two groups. Twenty-five patients underwent AA repair, and 25 underwent repair with an MO incision.

There were a total of 43 males and seven females in the study group. The average follow-up period was one year. The mean age was similar between the two: 44.32 years in the AA (range 22-62 years) and 46.32 years in the MO group (range 28-61 years).

The average rotator cuff tear size was 2.04 cm (range 1-4.5 cm). There was no statistically significant difference in tear size between the two groups (in AA 2.08 cm and in MO 2 cm, p-value of 0.263).

Among the 25 patients in the AA group, 18 had dominant side involvement, 15 had a history of trauma, and 16 were manual labourers, while among the 25 in the MO group, 16 had dominant hand involvement, 14 had a history of trauma, and 16 were manual labourers (Table 1).

**Table 1:** Characteristic of population in two groups

Study variables	All arthroscopy (AA) (n = 25)	Mini-open (MO) (n = 25)	P-value
Mean age (years)	44.32 (range 22-62)	46.32 (range 28-61)	0.072
Males	92%	80%	0.221
Dominant side	18	16	0.544
History of trauma	15	14	0.774
Manual labourer	16	16	1.000
Tear size (cm)	2.08	2	0.263
No. of anchors used	1.48	1.48	1.000
Time lag before surgery (weeks)	27.8	28.56	0.404
Duration of surgery (min)	89.68	57.24	0.017

Overall, there was a significant improvement at one-year follow-up from pre-operative status in shoulder function as measured by the UCLA score. The UCLA score improved pre-operatively from 13.92 to 29.76 post-operatively at the final follow-up. Additionally, the UCLA score was not statistically significant between the two groups (AA and MO). The VAS score for pain was measured on a continuous scale from 0 to 100. Pain was significantly decreased after surgery, as evidenced by the score, i.e., from a pre-operative score of 74.58 to a score of 21.38 one year post-operatively. The AA group was associated with statistically significant improvement in the VAS pain score at three and six months (p-value 0.024 and 0.026, respectively).

As for shoulder range of movement, active forward flexion and abduction improved from 93.4° and 66.1° pre-op to 145° and 115.9° one-year post-op, respectively. Passive external rotation from 38.3° to 71.8° and passive internal rotation from 25.6° to 46.8° also showed significant improvement at final follow-up when compared to status before surgery. There was no statistical significance in the range of movement when both groups were compared (Table 2).

**Table 2:** Mean changes in both groups ROM: range of motion; UCLA: University of California at Los Angeles; VAS: Visual Analogue Scale

Score and ROM	Pre-operative		3 months post-op		6 months post-op		One-year post-op	
	AA	MO	AA	MO	AA	MO	AA	MO
UCLA	13.96	13.88	25.84	24.64	28.36	27.92	30	29.52
VAS for pain	76.72	72.44	26	31.68	22.12	27.16	19.76	23
Active forward flexion	96.4°	90.4°	133.8°	129°	143.6°	137.2°	143.6°	146.4°
Active abduction	67°	65.2°	100.8°	102.2°	108.8°	110°	114.8°	117°
Passive external rotation	38°	38.6°	60.2°	62.2°	65.8°	67°	71.4°	72.2°
Passive internal rotation	24.8°	26.4°	38.2°	37.6°	42.6°	42.2°	47°	46.6°

In order to compare the results of the AA and MO rotator cuff repair groups, these two groups were analysed separately. When post-operative improvement was compared between the groups for the UCLA score, VAS score for pain, and active or passive glenohumeral motion, no significant difference was noted between the groups at a one-year follow-up (Table 3).

**Table 3:** p-Value comparison in both groups ROM: range of motion; UCLA: University of California at Los Angeles; VAS: Visual Analogue Scale

Score and ROM	Pre-operative	3 months post-op	6 months post-op	One-year post-op
UCLA	0.607	0.148	0.556	0.692
VAS for pain	0.187	0.024	0.026	0.218
Active forward flexion	0.723	0.449	0.323	0.290
Active abduction	0.237	0.395	0.257	0.552
Passive external rotation	0.495	0.809	0.428	0.878
Passive internal rotation	0.049	0.453	0.180	0.113

## Discussion

Symptomatic rotator cuff tears are a common cause of shoulder pain and impairment and can be grossly categorized as traumatic or degenerative in nature. In the initial stage, many rotator cuff tears may be managed conservatively with acceptable functional outcomes in the medium term, especially in elderly patients with lower functional demands [23]. However, surgical repair is often indicated in degenerative cases refractory to initial conservative management as well as acute traumatic tears in active patients [9].

Arthroscopy was first used in the treatment of rotator cuff tears as a diagnostic tool, enabling the surgeon to diagnose glenohumeral pathology [24]. Recent years have shown an increased interest in AA technique as arthroscopic techniques continue advancing and improving. Arthroscopy is technically more demanding than mini-open surgery. Nonetheless, the minimally invasive nature of arthroscopy has resulted in substantially lower post-operative pain levels, curtailed recovery time by early rehabilitation, and improved cosmetic results.

MO repair has the advantage of inspecting any intra-articular pathology and repairing the tendon with bone tunnels without insulting the deltoid origin, making it a popular technique. The MO repair provides direct visualization of the cuff muscles and allows the surgeons to place the stitches via an open technique, which might be more convenient and accessible for surgeons. This method also allows for tension-absorbing stitches to be placed in the cases where they are needed.

The primary finding of this study comparing AA and MO repair surgical techniques was the absence of a significant difference. Our study confirms that mid-term results for AA and MO rotator cuff repair are similar. However, patients do garner the benefits of treatment earlier (12 weeks) with the arthroscopic procedure. Shoulders in the AA group showed slightly better motion in the initial period at three months post-op. In addition, there was a significant decrease in VAS pain scores in the early phase in the AA group. These results are in agreement with Kang et al. [14], van der Zwaal et al. [25], and Walton and Murrell [26], whose study found earlier improvement in pain, better ROM, and greater satisfaction in the arthroscopy group. However, the functional outcome, pain, and ROM did not significantly differ between patients treated with AA repair and those treated with MO repair in the final follow-up in the first year after surgery. Therefore, the choice of surgical technique implemented for rotator cuff repair should not be simply based on decreased post-operative pain and initial improvement in ROM scores.

Repair of the rotator cuff via AA needs skilled techniques and requires a long learning period for surgeons to become specialists in this field. Besides, as only implantable suture anchor devices can be used in AA surgery, it also results in increased surgical time. Because the MO method has varied repair options, from bone tunnels to implantable suture anchors, it is presumably easy to learn and consumes less operative time.

Regarding post-operative rehabilitation, AA benefits MO repair because it induces less soft-tissue damage. Despite preserving the deltoid origin, the MO method still requires a split in the deltoid fibres extending into the subdeltoid bursa to expose the operative region, which may result in subacromial scarring and stiffness [27]. The two most common complications of rotator cuff repair using these two methods are adhesive capsulitis and re-tear. Our study compared the two groups and found no differences in the incidence of re-tear.

This study supports the continued use of the arthroscopic repair technique or mini-open technique based on the surgeon’s experience, the infrastructure available, and the financial constraints of the patient. The surgical outcome is mainly dependent on the size of the tear and not on the method of repair [13,17]. Rotator cuff surgery aims to eliminate pain and reintegrate its function. Although there are many surgical techniques related to rotator cuff tears, there is no definite agreement about the superiority of a given technique [13,14,18,19,24]. The results did not define a technique as being superior or coherent with literature. The limitations of our study were that it was a single institution setting, there was a relatively small number of patients, and the follow-up period was not very long-term.

## Conclusions

The present study found that the AA approach was associated with less pain, a lower VAS pain score, and a better range of movement during the early recovery period. However, there were no differences in clinical and functional outcomes between the AA and MO procedures in the final review or incidence of complications. Both methods are effective and viable options for surgeons to repair rotator cuff tears, and both approaches are equivalent regarding the long-term outcomes. The choice of operating technique depends upon the surgeon's expertise and comfort level.
